# Benefits of Equine-Assisted Therapies in People with Multiple Sclerosis: A Systematic Review

**DOI:** 10.1155/2022/9656503

**Published:** 2022-04-27

**Authors:** Ana Myriam Lavín-Pérez, Daniel Collado-Mateo, Alejandro Caña-Pino, Santos Villafaina, Jose Alberto Parraca, María Dolores Apolo-Arenas

**Affiliations:** ^1^Centre for Sport Studies, Rey Juan Carlos University, 28943 Fuenlabrada, Madrid, Spain; ^2^Department of Medical Surgical-Therapy, Medicine Faculty, Extremadura University, 06006 Badajoz, Spain; ^3^Faculty of Sport Sciences, University of Extremadura, Cáceres, Avenida de la Universidad s/n, Cáceres 10003, Extremadura, Spain; ^4^Departamento de Desporto e Saúde, Escola de Saude e Desenvolvimento Humano, Universidade de Évora, Évora, Portugal; ^5^Comprehensive Health Research Center (CHRC), University of Évora, Évora, Portugal

## Abstract

This systematic review aimed to provide an up-to-date analysis of the effects of equine-assisted therapies (EAT) in people with multiple sclerosis (PwMS). The Preferred Reporting Items for Systematic Reviews and Meta‐Analyses (PRISMA) guidelines were followed to conduct this systematic review. PubMed and Web of Science databases were employed in the search, which ended in February 2022. The risk of bias analysis was performed using the Evidence Project tool. After removing duplicates, thirty-nine studies were identified. However, only ten fulfilled the inclusion criteria and were included in this systematic review. Therefore, a total of 195 PwMS, aged between 40.3 and 51.3, were included in this systematic review. EAT-based interventions had a mean length of 13.6 weeks with a session´s frequency ranging from ten to once a week. All sessions involved real horses and lasted a mean of 34.4 min. Among the included articles, four were randomized controlled trials (RCT), four did not perform randomization, and two employed a prepost design without a control group. RCTs showed positive effects on quality of life, fatigue, balance, spasticity, and gait speed. Furthermore, non-RCT showed improvements in balance, spasticity, and postural control (postural control was not assessed in RCT studies). Importantly, significant effects were only observed when the comparison group was inactive or followed usual care. Therefore, EAT is a promising and effective therapy to improve quality of life, fatigue, balance, spasticity, and gait speed in PwMS. However, since comparison groups are heterogeneous, results could vary depending on the research design. Moreover, the inclusion of noncontrolled studies (in order to have a wide perspective of the state of art) could increase the risk of bias and make the results be taken with caution.

## 1. Introduction

Multiple sclerosis (MS) is a chronic disease characterized by a progressive demyelination and an axonal loss across the central nervous system. MS symptoms can be manifested singly or combined and include several manifestations such as fatigue, paraesthesia, stiffness, muscle spasms, tremors, weakness, dizziness, gait disturbance, or pain [1]. These symptoms significantly reduce the health-related quality of life (HRQoL) of people with MS (PwMS) [2, 3]. Furthermore, previous studies have shown that PwMS often showed less postural control and, consequently, a higher risk of falling [4, 5]. This is relevant, since falls are associated with injuries, lower participation, and increased fear of falling in this population [6]. Despite the benefits of pharmacological and nonpharmacological treatments, rehabilitation programs are encouraged to improve both physical health and mental health of PwMS.

Complementary and alternative treatments emerged to reduce the severity of symptoms and to enhance the HRQoL of PwMS. However, evidence about the efficacy of these therapies has sometimes been questioned [7]. In this regard, complementary and alternative therapies have been included in less than half of the clinical practice guidelines for PwMS [8]. Therefore, there is a need to review studies that provide more evidence and clarify which therapies should be recommended to reach higher benefits and reduce the symptoms' limitations [8].

Previous studies in the field of animal-assisted interventions reported positive benefits in mental and physical health [9, 10]. Equine-assisted therapies (EATs), which are part of animal-assisted intervention, have shown positive benefits in older adults [11], autism [12], children with attention-deficit/hyperactivity disorder [13], cerebral palsy [14], or chronic pain populations [15]. The physical mechanism underlying the physical benefits of riding a horse is related to the rider's movement induced by the horse during walking. This movement pattern has some similarities with the human gait, generating a bilateral and continuous stimulus that leads to voluntary and involuntary muscular activity. This pattern has been shown to help improve or maintain control of posture balance [16]. Furthermore, some psychological benefits have also been observed after EAT, including improvements in self-esteem, self-regulation, HRQoL, competency, emotional wellbeing, and social support [17, 18].

A previous review, in 2010, aimed to systematically review the evidence for hippotherapy as a therapy to improve balance [19]. Although only three studies were included in that review, the authors concluded that hippotherapy has a positive effect on balance in PwMS. However, due to the variety of terms used to inadequately refer to EAT (such as equine-assisted therapy, horse-riding, horseback riding, or therapeutic riding), we believed that further evidence might not have been included. Furthermore, no other review has been conducted specifically to evaluate the effects of EAT (including more search terms related to EAT) or to evaluate other health-related variables in PwMS such as fatigue, HRQoL, or walking performance. Therefore, the current systematic review aimed to provide an up-to-date analysis of the effects of EAT in different health-related variables of PwMS.

## 2. Methods

This systematic review was performed according to the Preferred Reporting Items for Systematic Review and Meta-analysis (PRISMA) guidelines [20]. This study was registered in the International Prospective Register of Systematic Reviews (PROSPERO) with the following identification number: CRD42020220433.

### 2.1. Data Sources and Search Strategy

PubMed and Web of Science databases (including Current Contents Connect, Derwent Innovations Index, Korean Journal Database, Medline, Russian Science Citation Index, and SciELO Citation Index) were used to identify potential studies.

A wide variety of terms describing equine-assisted interventions can be found regarding context, country, or type of intervention such as hippotherapy, equine-assisted intervention, or therapeutic horseback riding. This represented a huge source of confusion, since these terms are used interchangeably [21]. Therefore, taking this variety into account, we have followed the definition of EAT made by the Professional Association of Therapeutic Horsemanship International (PATH) to include and analyze the articles. In this regard, EAT has been considered as an intervention that can incorporate equines or equine environments for rehabilitation goals directed by professionals in the field. Thus, the following search string was employed: (“multiple sclerosis”) AND (“hippotherapy” OR “equine-assisted” OR “horse-riding” OR “horseback riding” OR “therapeutic riding”). Year, type of design, and language restrictions were not applied in the search. The search process ended in February 2022. Duplicated studies were excluded and articles' titles, abstracts, and full texts were carefully screened by two of the authors (A.M.L-P. and A.C.P.).

Studies were included in the systematic review if they fulfilled the following criteria: (1) participants suffered from MS; (2) the article analyzed the effects of EAT on physical or mental health-related outcomes; (3) the study conducted an EAT intervention; and (4) they were randomized or nonrandomized controlled trials with prepost data. Moreover, the studies were excluded when: (1) they were written in a different language from English or Spanish; (2) they were a review, study protocol, conference abstract, or a case report; (3) the article was not focused on PwMS.

The study selection was performed by one author, A.C.P., and checked by another, A.M.L-P. Disagreements between these authors were solved through discussion with D.C-M.

### 2.2. Risk of Bias Assessment

The Evidence Project tool [22] was employed to evaluate the risk of bias of the selected studies. This tool is composed of eight items that cover study design, the participants' representativeness, and the equivalence of comparison groups. In this regard, the study design includes items referring to cohort, control, or comparison group and prepost intervention data. Participants´ representativeness includes items that analyze the random assignment of participants to the intervention, random selection of participants for assessment, and follow-up rate of 80% or more. Lastly, the comparison groups' equivalence is assessed with items concerning the equivalent on sociodemographics and the equivalent at baseline. This scale allows evaluating both randomized and nonrandomized trials.

### 2.3. Data Extraction

According to PRISMA methodology [20], participants, intervention, comparison treatments, outcomes, and study design (PICOS) data were extracted. Accordingly, information concerning participants' characteristics, study design, sample size, age, years from MS diagnosis, disability level, and body composition were exported from each article. Moreover, intervention characteristics such as intervention length, treatment frequency, duration of the sessions, setting where the EAT was carried out, type of exercise performed, and its description were analyzed. For each variable the “pre-,” “post-,” and the change between pre- and post- data were extracted, reporting means and standard deviations as well as the reported effects (within- and between-group differences) for each article. The extraction process was conducted by two authors (A.M.L-P. and A.C.P.). Regarding the reported data, specific statistical calculations were performed according to each trial design. First, the Standardized Mean Differences between control and equine-assisted therapy groups were calculated by utilizing the ReviewManager Software (RevMan, 5.3) [23]. The selected method was the inverse variance with random effects and a 95% confidence interval (CI) [24]. In randomized control trials (RCT), results were calculated taking into account the data after intervention. Meanwhile, in non-RCT, changes from baseline results were the data selected. Moreover, in that non-RCT without enough change from baseline data, Cohen's effect size and its corresponding confidence interval (95%) were calculated [25].

## 3. Results

### 3.1. Study Selection

**Figure 1:**
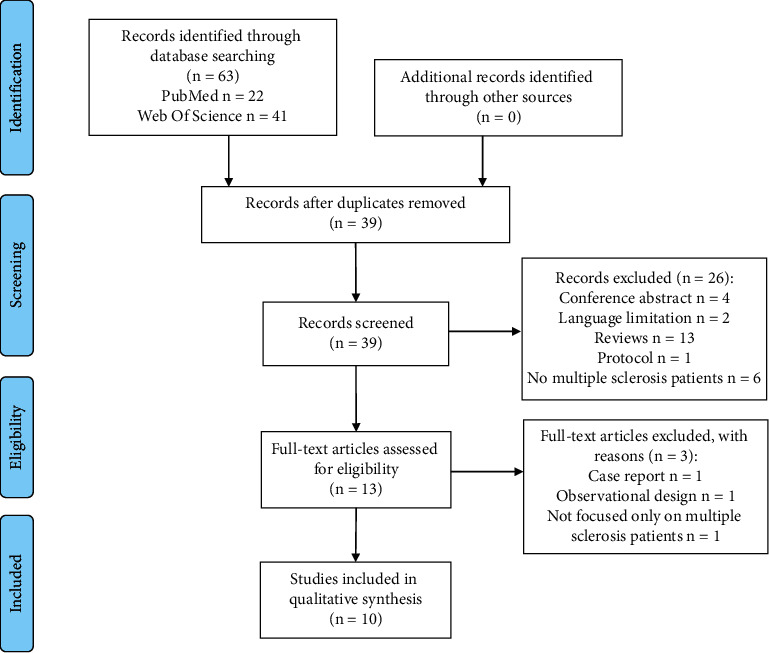
Flow diagram of the study selection.

A total of 63 publications were identified in the electronic databases: 22 studies in PubMed and 41 in the Web of Science. After removing duplicates, 39 studies were screened by reading the title and abstract. Twenty-six studies were excluded because they were reviews (thirteen studies), conference abstracts (four studies), protocols (one study), not focused on PwMS (six studies), or not written in English or Spanish (two studies). Thirteen studies were assessed for eligibility. However, three studies did not fulfil the inclusion criteria, since one was a case report, one was an observational study, and one included another type of disease apart from MS. Therefore, our systematic review included ten studies (Figure 1).

### 3.2. Characteristics of the Participants

Table 1 shows the study design, sample size, age, years from MS diagnosis, disability level, and body composition for each article. A total sample size of 195 participants was included in this systematic review. 104 participants were included in the equine-assisted therapy group (EATG) and 91 in the CG, of which 63 were inactive participants and 28 performed a different type of intervention. The mean age was 46.2 years in the EATG (from 41.3 to 47.9) and 45.6 years in the CG (from 40.3 to 51.3). Median and interquartile range were reported by Vermöhlen et al. (2017). The EATG was diagnosed with MS with a mean symptom duration of 11.6 years (from 8.3 to 22.3 years) and the CG was diagnosed for 10.8 years (from 8.2 to 17.5 years).

As for participants´ disability levels, the Extended Disability Status Scale (EDSS) [26] was used in four studies. This scale ranges from 0 to 10 in 0.5 units. Higher scores represent higher levels of disability. For instance, scores of 1 to 4.5 refer to people who can walk without aid and scores higher than 5 referred to people who suffered from walking impairments. Participants included in this systematic review had an EDSS score that ranged from 1 to 5.2. Nevertheless, one study included participants with EDSS higher than 5.

### 3.3. Characteristics of the Interventions

Table 2 depicts the intervention length, treatment frequency, duration of the sessions, setting where the EAT was carried out, type of exercise performed, and its description for each article. The EAT interventions assessed in this systematic review had a mean length of 13.6 weeks with a standard deviation of 5.6. Four interventions performed the treatment twice a week [27–31] and four performed the treatment once a week [28, 32–34], while one conducted the intervention ten times per week [35]. EAT sessions lasted a mean of 34.4 min with a standard deviation of 8.9. However, some of the interventions made a progression in the sessions' duration throughout the program [30, 32, 36]. Two studies did not describe the characteristics of their intervention.

Regarding sessions characteristics, most of the EAT interventions performed a warm-up and a cool-down with a duration from 5 [28, 29, 31, 34] to 10 minutes [36], based on upper and lower extremities stretching or slow walking while connecting with the horse. EAT exercises (with a mean duration of 30 minutes) were focused on balance, mobility, changes in direction and speed [29, 31, 34, 35], progressive difficulty of tracks [28, 29] based on riders' motor skills [32], and postural control on the horse by the employment of different riding techniques [36].

EATG participants were also enrolled in physiotherapy sessions in only one study as its CG [32]. Also, Frevel et al. (2015) and Menezes et al. (2013) included active CGs, performing home-based exercises focused on balance, postural control, strength [30], pilates, swimming, or weightlifting [36]. The rest of the studies, except two [31, 35], included CGs that followed standard care routines [27–29, 32, 33].

In relation to EAT safety, only one article [27] reported that one participant fell off the therapy horse and was able to continue therapy. Moreover, two participants experienced, at the beginning of the intervention, an MS relapse accompanied by painful muscle contractions. The other two articles employed two side-walkers in order to reduce risks [29, 34].

### 3.4. Health Variables Evaluated

Different health outcomes were assessed before and after the interventions. HRQoL was studied using King's Health Questionnaire (KHQ) [33], the Multiple Sclerosis Quality of Life-54 (MSQOL-54) [33] and its mental and physical health subscales [27], the Short Form 36 (SF-36) and its dimensions [35], the Hamburg Quality of Life Questionnaire in Multiple Sclerosis (HAQUAMS) [30], and the Functional Assessment of Multiple Sclerosis Quality of Life (FAMS). [28]. Four studies analyzed fatigue using the Fatigue Severity Scale (FSS) [27, 28, 30], the Fatigue Impact Scale (FIS) [33], and the Modified Fatigue Impact Scale (MFIS) [27, 28]. Five articles analyzed balance [27, 30, 31, 34, 35] using the Berg Balance Scale for static balance [27, 30, 31, 34, 35] and the Dynamic Gait Index (DGI) for dynamic balance [30]. Moreover, four studies assessed the benefits in mobility using the Performance Oriented Mobility Assessment (POMA) [32, 34] and the Timed Up and Go test [28, 35] and two articles studied the spasticity through the Numeric Rating Scale (NRS) [27] and the Modified Ashworth Scale [33]. Walking performance was evaluated using the 6-minute walking test [29] and the 2-minute walking test [30]. Regarding gait performance analysis, three studies analyzed the gait speed [29, 32] and the Functional Gait Assessment (FGA) [31] after the EAT interventions. Lastly, the following variables were analyzed in a single study: disability, with the Extended Disability Status Scale [32]; pain, with Visual Analogue Scale [27]; mobility and performance in activities of daily living with the Barthel Index [32]; and postural control with the evaluation of the center of pressure during static balance tasks [28, 31, 36] and the Sensory Organization Test (SOT) [35].

### 3.5. Effects of Equine-Assisted Therapy on HRQoL and Physical Outcomes

Table 3 summarizes the results in HRQoL, fatigue, balance, mobility, spasticity, walking performance, gait performance, disability, pain physical functioning, and postural control. Regarding HRQoL, significant within-group differences were found in two articles [28, 33], whereas Hammer et al. (2005) did not find significant differences. Between-group differences were observed in both physical and mental health dimensions (*p* < 0.001) [27]. However, when comparing EAT to Internet-based home training, Frevel et al. (2015) did not find significant differences [30].

As for participants' fatigue perception, when the effects of EAT were compared to an inactive CG, significant between-group differences emerged (*p*=0.002 [27] and *p*=0.017[28]). In contrast, when the CG performed Internet-based home training, significant differences were not reached [30]. Moreover, three of the studies found a significant decrease in fatigue in the EATG after the intervention (*p* < 0.001 [28, 33] and *p* < 0.05 [30]).

Results in physical outcomes showed that static balance significantly improved after EAT intervention in comparison to an inactive CG (*p*=0.047 [26v] and *p*=0.04 [34]). However, Frevel et al. (2015) did not find significant between-group differences (EATG versus home-based training group); however, significant within-group improvements were found in the EATG [30]. In this regard, Lindroth et al. (2015) did not find differences after the EAT intervention in balance [31].

With respect to mobility, significant between-groups differences were not found. However, two studies showed significant within-group results in mobility [28, 34]. Spasticity results showed that EAT interventions could significantly reduce this aspect when compared to the CG [27, 33]. Considering walking and gait performance, Moraes et al. (2020) revealed significant differences between (*p* < 0.001) and within (*p* < 0.001) groups in all the tests performed (6MWT, gait velocity, and gait cadence) [29]. However, Flevel et al. (2015), in walking performance, and Muñoz-Lasa et al. (2011), in walking performance and gait, did not show positive results [30, 32]. Regarding postural control, Menezes et al. (2013), Moraes et al. (2021) and Lindroth et al. (2015) showed that EAT can improve the amplitude of the anterior-posterior center of pressure (*p* < 0.01), mean medial-lateral velocity (*p*=0.02), and the speed of the center of pressure in stable and foam superficies (*p* < 0.001) [28] and a positive trend was observed in the SOT [31]. Concerning activities of daily living, pain, or physical independence, EAT interventions did not show significant benefits when compared to the CG [27,32].

To summarize, between-groups differences were only observed when comparing EAT with an inactive CG [27–29, 33, 34, 36]. Therefore, between groups, significant differences were not observed when EAT was compared to an active CG [30, 32].

### 3.6. Risk of Bias

The mean score of the risk of bias analysis with the Evidence Project tool was 5.7 out of 8 with a standard deviation of 1.57 and scores ranged from 3 to 7 (Table 4). Higher scores corresponded to RCT studies (7/8) [27–30] where assignment to experimental groups was randomized. Item-by-item analysis showed that assessment of the quality of the study design (items 1 and 3) was satisfactorily reached by all the studies. However, in the participants' representativeness evaluation, more heterogeneous results were found. Item 4, which assessed the “random assignment of participants to the intervention,” was only fulfilled by three studies, while item 5 (“random selection of participants for assessment”) was not reached for any of the studies, whereas all the studies positively scored item 6 (“follow-up rate of 80% or more”). Besides, in the equivalence of comparison groups, except for three studies on item 8 (referred to the “comparison groups equivalent at baseline on outcome measures”) and two studies in item 7, all the studies fulfilled the requirements. However, the total scores and the section analysis were influenced in their low results by the inclusion of two studies with no CG [31, 35].

## 4. Discussion

This systematic review analyzed the effects of EAT in PwMS. Ten articles were included in this systematic review: three were RCTs, and seven did not perform randomization. RCTs showed positive effects of EAT on HRQoL, fatigue, balance, spasticity, and gait speed. Furthermore, non-RCTs showed improvements in balance, spasticity, and postural control (postural control was not assessed in RCTs studies). Importantly, significant effects were only observed when the comparison group was inactive or followed their usual care. Furthermore, taking into account the fact that non-RCTs studies are more prone to bias and the heterogeneity among the selected studies (mainly due to the inclusion of articles focused on different health-related outcomes and articles with active and inactive CGs), results might be taken with caution.

Results of this systematic review showed that significant improvements can be reached with EAT when comparing its effects to an inactive CG or a CG that followed usual care. This is congruent with previous studies that showed the potential of physical activity to increase the HRQoL and physical function of PwMS [27, 37, 38]. Thus, physical activity is considered a useful therapy against MS-related impairments when EAT cannot be performed. This is relevant, since EAT is usually expensive due to animal care or displacement. Thus, previous studies have analyzed the effects of horse-riding simulators in special populations [11, 39]. These simulators mimic horse movements, leading to postural responses [38] with fewer costs than real horses [40]. However, the emotional response of riding a horse [41], even the temperature (higher than humans which could have a beneficial effect on spasticity), or the outdoor environment could add some benefits to EAT [17, 42]. Thus, future studies should try to isolate the effects of EAT.

Positive results were found in the HRQoL [27, 28, 33] and fatigue [27, 28, 30, 33] after EAT in PwMS. The observed improvements could be considered clinically significant, since a difference of at least 0.45 points on the FSS or 4 points on the MFIS has been reached [43]. Fatigue is one of the main causes of impaired HRQoL among PwMS, although it is poorly understood [44]. However, different mechanisms, including proinflammatory cytokines (TFN-*α*), endocrines influences, and axonal loss, have been proposed [44]. TFN-*α* mRNA expression is increased among PwMS with fatigue [45, 46]. In this regard, previous studies have shown that animal-assisted intervention had hormonal effects, such as an increase in oxytocin release. This is quite relevant, since a previous study showed that oxytocin treatment decreases TFN-*α* [47]. Thus, future studies should explore if fatigue reduction after animal-assisted intervention could be related to oxytocin releases.

Regarding HRQoL, benefits could be related to the improvement of physical function (balance, postural control, mobility, walking, and gait performance). However, both the physical and the mental dimensions of the MSQoL-54 have been improved, reaching the minimum clinically important difference [27]. Therefore, the significant increase in the mental dimension of MSQoL, and the total HRQoL with the FAMS questionnaire [28], indicated that changes were not limited to physical benefits. Furthermore, other benefits such as the reduction of fatigue [28, 30, 33] or pain [15, 27] may have a significant impact on HRQoL. However, controversial results were found, since Frevel et al. [30] only reported improvements in the cognition, lower limb, and mood subscales but not in the overall score of the Hamburg Quality of Life Questionnaire in Multiple Sclerosis. These disagreements in results may be explained by the use of different tools to assess HRQoL, which cover different dimensions that influence the final score [58, 59]. Nevertheless, taking into account the obtained results and the large impact of MS on HRQoL, EAT could be a potential therapy to enhance not only the physical dimension of the HRQoL, but also the mental dimension. This can be considered one of the major findings of the current review.

In relation to the physical effects of EAT interventions on PwMS, there is consensus among the analyzed studies showing benefits on balance. These changes can be considered clinically important, since Berg Balance Scale increased more than three points [51]. Nevertheless, taking into account the *p* values, significant between-group differences were observed when the group that received EAT was compared to an inactive CG [27, 34]. In this way, only within-group differences were observed in balance, postural control, and strength when EAT intervention was compared with a group that performs Internet-based home training [30]. Moreover, previous studies in the field of EAT have shown significant benefits in trunk/head stability [15, 47, 52–54], which positively influences balance [27, 30, 34]. This is relevant, since improvements in balance can considerably reduce the risk of falling [30, 53, 55], being a major limitation in PwMS [6]. Therefore, according to the results summarized in this systematic review, a 30-minute session per week for 12 sessions may be enough to achieve improvements in balance [27], since the improvements are found when the EAT was received once a week [27, 34], like when there were twice [30].

Mobility and walking performance of PwMS improved after EAT interventions [29, 30, 32, 34]. Improvements in walking performance may be related to the reduction in the stance time and double support time as well as the increase in balance time [29]. Benefits in gait parameters after EAT may be due to a physical stimulus induced by the movement of the horse. In this regard, horses perform a three-dimensional rhythmic movement, being patients' pelvis movements similar to the movement produced during human gait. Thus, riding a horse leads to bilateral, continuous, and symmetrical movement patterns that stimulate muscle fibers and positively affect the control of posture and balance [43]. Furthermore, its practice requires the participation of the whole body and, therefore, it contributes to changes in muscle tone and motor coordination [56]. For this reason, this type of therapy has been used as a complementary strategy to reduce spasticity and improve motor skills [32].

This systematic review has some limitations. First, only studies in Spanish and English were included. Second, due to the heterogeneity of the studies included in the systematic review (in terms of interventions, CGs, participants, and outcomes), a meta-analysis was not possible. Concerning participants' heterogeneity, only four of the ten studies included in this systematic review used a scale to evaluate the disability level. Thus, future studies should incorporate specific scales for PwMS, such as EDSS, to characterize the participants. Third, some studies were not randomized, which could have affected the obtained results due to an increase of risk of bias in these studies. Therefore, RCTs with homogeneous populations are encouraged to assess the effect of EAT in PwMS to ensure that the groups are equivalent at baseline. Lastly, only one article detailed the side effects of this therapy [27] and two studies reported safety strategies to reduce risks [30, 34]. Future studies are encouraged to detail any side effects detected or to report that no side effects were identified.

## 5. Conclusion

This systematic review is the first to evaluate the benefits of EAT on PwMS. Promising and positive results were achieved for HRQoL, fatigue, balance, and gait. However, large heterogeneity was also observed between the included studies. Thus, more RCTs are needed to evaluate the effects of EAT on those variables.

## Figures and Tables

**Table 1:** Characteristics of the participants included in the systematic review.

Study	Randomization	Group	Sample size (% of females)	Age (SD)	Years from diagnosis (SD)	Disability level (SD)	Body composition data (SD)
*Moraes (2020) and Moraes (2021)*	Yes (RCT)	EATG	*n* = 17 (94.18%)	45.5 (9.7)	9 (6.1)	EDSS (median): 2	H: 162 (4.2)/W: 67 (13.1)/BCD: 25.5
		ICG	*n* = 16 (93.75%)	44.8 (8.8)	8.8 (5.7)	EDSS (median): 1.75	H: 163 (6.6)/W: 68.7 (13.4)/BCD: 25.9
*Muñoz-Lasa (2019)*	No (non-RCT)	EATG	*n* = 6 (50%)	41.3 (3.3)	15.5 (5)	NR	NR
		ICG	*n* = 4 (25%)	51.3 (4.6)	17.5 (7.3)	NR	NR
*Vermöhlen (2017)*	Yes (RCT)	EATG	*n* = 30 (90%)	50 (median); *R* (45–53)	16.5 (median) *R* (11–20)	EDSS < 5 : 10 EDSS ≥ 5 : 20	W: 67 (10.3)
		CG	*n* = 37 (72.97%)	51 (median); *R* (47–56)	17.6 (median) *R* (11–27)	EDSS < 5 : 11 EDSS ≥ 5 : 26	W: 70.6 (9.9)
*Frevel (2015)*	Yes (RCT)	EATG	*n* = 9 (88.8%)	46.9 (7.6)	22.3 (8.3)	EDSS: 3.8 (1.1)	H: 167.9 (9.0)/W: 72.5 (12.3)/BCD: 25.7
		ACG	*n* = 9 (77.77%)	44.3 (8.1)	16.1 (11.3)	EDSS: 3.8 (1.5)	H: 168.2 (8.2)/W: 65.0 (8.9)/BCD: 23.03
*Lindroth (2015)*	No (non-RCT)	EATG	*N* = 3 (66.66%)	52.33 (13.28)	14 (13.89)	4 (1.32)	NR
*Menezes (2013)*	No (non-RCT)	EATG	*n* = 7 (85.71%)	44 (9.1)	8.6 (9.6)	NR	H: 162 (1)/W: 68.1 (16.6)/BCD: 25.9 (5.3)
		ACG	*n* = 4 (50%)	40.3 (15.9)	8.2 (5.6)	NR	H: 142 (6.2)/W: 72 (34.2)/BCD: 25.2 (25)
*Muñoz-Lasa (2011)*	No (non-RCT)	EATG	*n* = 12 (58.33%)	44.8. Range: 34–59	8.3 (7)	EDSS: 5.2 (1.2)	NR
		ACG	*n* = 15 (60%)	46.2. Range: 38–64	7.8 (7)	EDSS: 4.9 (1.3)	NR
*Silkwood-Sherer (2007)*	No (non-RCT)	EATG	*n* = 9 (55.55%)	42.4 (14.2)	9.9 (8.2)	NR	NR
		ICG	*n* = 6 (66.66%)	47.7 (14.1)	12.7 (6.6)	NR	NR
*Hammer (2005)*	No (non-RCT)	EATG	*n* = 11 (81.8%)	47.9 (8.4)	10 (7)	5 (6)	NR

EATG: equine-assisted therapy group; ICG: control group; NR: not reported; SD: standard deviation; EDSS: Extended Disability Dtatus Scale; H: height (cm); W: weight (kg): BCD: body composition data; R: range; ACG: active control group.

**Table 2:** Characteristics of the interventions included in the systematic review.

Study	Group	Duration	Frequency	Sessions´ duration	Setting	Type of exercise	Exercise description
Moraes (2020) and Moraes (2021)	EATG	8 weeks	2 days/week	35 min	Military Police Hippotherapy Center in Brasilia, Brazil	Hippotherapy	Stretching and warming-up exercises on the horse (5 min); EAT (28 min): balance, mobility, and functional exercises. Thirteen progressive tasks were performed (from “serpentine movement throwing hoops on cone” to “short obstacle courses”); calm down (2 min): relaxation with the horse always in motion.
	ICG	Normal therapeutic routine and participating in the intervention after the follow-up evaluation					
Muñoz-Lasa (2019)	EATG	24 weeks	1 day/week	From 20 to 40 min	MHG foundation equestrian therapy team	Hippotherapy	NR
	ICG	NR					
Vermöhlen (2017)	EATG	12 weeks	1 day/week	30 min (EAT)	Multicenter (five centers in Germany)	Standard care + horseback riding therapy	Developed following the guidelines for hippotherapy of Deutsches Kuratorium für Therapeutisches Reiten.
	ICG	Standard care: symptomatic drug treatment, immunotherapy, and physiotherapy					
Frevel (2015)	EATG	12 weeks	2 days/week	20–30 min	Hospital with therapeutic riding center “Gut Üttingshof, Bad Mergentheim”	Hippotherapy	EAT: riding forward, backward, side-ways, changes in horse´s speed from slow to moderate, diagonal change of direction, sudden stops and starts. Balance exercises and movements on the horse: trunk rotations exercises and arm lifting with eyes open and closed (lying on the horse´s back with the front or rear, sitting side-ways or backward).
	ACG	12 weeks	2 days/week	45 min	Internet-based home training	Balance, postural control, and strength exercises	Balance, postural control, and strength exercises for the main muscle groups of the lower limbs, trunk, and shoulder girdle. 8–15 repetitions. 2–3 sets of moderate intensity (Borg Scale: 11–14).
Lindroth (2015)	EATG	6 weeks	2 days/week	40 min	Adaptive riding center	Hippotherapy	5-min warm-up with the participant sitting forward on the horse with no stirrups, followed by 30 min of individualized intervention (balance exercises in different positions) and a 5-min cool-down.
Menezes (2013)	EATG	16 weeks	2 days/week	50 min	Association of PwMS of Santa Maria, Brazil	Hippotherapy	Stretching and contact with the horse (10 min). Horse walking (30 min): use of different driving techniques and postures with postural control. The level of difficulty was gradually increased. Cool-down (10 min): exercise and stretching.
	ACG	Complementary therapeutic intervention: pilates, swimming, and weightlifting					
	EATG	20 weeks on and 4 weeks off in between	1 day/week	30–40 min (EAT)	Fundación Caballo Amigo in Villanueva de la Can˜ada (Madrid)	Therapeutic horseback riding and conventional physiotherapy	EAT: exercises based on rider's motor skills, balance, and body posture in a slow steady horse gait (four-beat walk). Physiotherapy: aerobic, balance, strength, and flexibility exercises.
Muñoz-Lasa (2011)	ACG	20 weeks on and 4 weeks off in between	1 day/week	40 min	ADEMM in Madrid	Conventional physiotherapy	Aerobic, balance, strength, and flexibility exercises.
Silkwood-Sherer (2007)	EATG	14 weeks	1 day/week	40 min	NR	Therapeutic horseback riding	Warm-up (5 min): slow pace (90–100 steps/min) and stretching on the horse, progressively increasing to moderate pace (125–130 steps/min). Individualized EAT (30 min): trunk rotations in sitting forward position, anticipatory challenges, change of direction exercises, sudden stops and starts, and speed changes from slow pace to trot (150 steps/min). Cool-down (5 min): stretching and moderate pace.
	ICG	NR: no rehabilitation program with the chance of participating in the intervention after the follow-up evaluation					
Hammer (2005)	EATG	11 weeks	10 sessions/week	30 min	NR	Therapeutic riding	Trunk rotation exercises, for example, reaching the ears or tail of the horse with one hand, reaching the opposite knee or diagonally, towards the ceiling. The exercises involved balance and driving skills.

EATG: equine-assisted therapy group; ICG: inactive control group, EAT: equine-assisted therapy; NR: not reported; ACG: active control group; PwMS: people with multiple sclerosis; EDSS: Extended Disability Status Scale.

**Table 3:** Effects of equine-assisted therapy intervention on the assessed instrument.

Study	Tool	Groups	Before intervention	After intervention	Effect size [CI 95%]	Differences (*p* value)	Between-group
			Mean (SD)	Mean (SD)		Within-group	
*HEALTH-RELATED QUALITY OF LIFE*							
Moraes (2021)	FAMS (total)	EATG	133.4 (35.2)	151.4 (38.3)	0.11 [−0.57, 0.80]^SMD^	*p* < 0.001	*p*=0.283
		ICG	143.2 (27.2)	138.7 (27.2)		*p*=0.360	
Muñoz-Lasa (2019)	KHQ	EATG	34.83 (16.15)	17.7 (6.95)	1.38 [ 0.1, 2.6]^d^	*p*=0.033	NSD
		ICG	32.36 (19.99)	34 (21.73)	−0.08 [−1.5, 1.3]^d^	NR	
	MSQOL-54 (2,3)	EATG	2.85 (0.9)	3.55 (0.61)	−0.85 [−2.0, 0.3]^d^	*p*=0.011	NSD
		ICG	2.63 (1.3)	2.61 (1.05)	0.02 [−1.4, 1.4]^d^	NR	
Vermöhlen (2017)	MSQOL-54 (physical health)	EATG	46.0 (14.2)	57.0 (15.1)	0.36 [−0.12, 0.85]^SMD^	NR	*p* < 0.001
		ICG	53.7 (14.6)	51.3 (15.9)		NR	
	MSQOL-54 (mental health)	EATG	62.6 (18.0)	75.7 (15)	0.64 [0.14, 1.13]^SMD^	NR	*p* < 0.001
		ICG	67.1 (17.2)	64.2 (19.9)		NR	
Frevel (2015)	HAQUAMS total	EATG	13.6 (2.3)	14.5 (2.1)	0.38 [−0.62, 1.37]^SMD^	NSD	NSD
		ACG	11.5 (4.1)	13.0 (4.9)		NSD	
Hammer (2005)	SF-36 (general health)	EATG	54.5 (25.82)	53.8 (19.26)	NA	NA	NA
	SF-36 (mental health)	EATG	70 (19.74)	69.6 (30.53)	NA	NA	NA
	SF-36 (physical functioning)	EATG	40 (24.30)	37 (20.38)	NA	NA	NA

*FATIGUE*							
Moraes (2021)	FSS	EATG	5.0 (1.6)	4.0 (1.7)	−0.17 [−0.86, 0.51]^SMD^	*p* < 0.001	*p*=0.335
		ICG	4.5 (1.7)	4.3 (1.7)		*p*=0.455	
	MFIS Total	EATG	44.2 (19.0)	32.3 (18.5)	−0.86 [−1.57, −0.14]^SMD^	*p* < 0.001	*p*=0.017
		ICG	48.1 (10.3)	45.9 (11.5)		*p*=0.461	
Muñoz-Lasa (2019)	FIS	EATG	5.52 (0.67)	1.93 (0.83)	4.76 [2.5, 7.0]^d^	*p* < 0.001	NSD
		ICG	2.85 (0.48)	2.87 (0.74)	−0.03 [−1.4, 1.4]^d^	NR	
Vermöhlen (2017)	FSS (sum score of FSS)	EATG	51.80 (10.5)	42.6 (11.4)	−0.38 [−0.86, 0.11]^SMD^	NR	*p*=0.002
		ICG	47.8 (11.9)	46.8 (10.6)		NR	
Frevel (2015)	FSS	EATG	5.40 (0.80)	4.4 (1)	0.24 [−0.75, 1.22]^SMD^	*p* < 0.05	NSD
		ACG	4.50 (1.90)	4.00 (2.00)		NSD	
	MFIS	EATG	44.7 (18.3)	22.9 (11.8)	−0.31 [−1.30, 0.67]^SMD^	*p* < 0.05	NSD
		ACG	34.6 (22.6)	28.5 (20.8)		NSD	

*BALANCE*							
Vermöhlen (2017)	BBS	EATG	40.60 (11.50)	47.0 (8.7)	0.19 [−0.29, 0.67]^SMD^	NR	*p*=0.047
		ICG	42.10 (10.90)	45.10 (10.90)		NR	
Frevel (2015)	BBS	EATG	40.30 (9.80)	45.8 (8.3)	−0.08 [−1.06, 0.90]^SMD^	*p* < 0.05	NSD
		ACG	43.50 (9.90)	46.50 (9.00)		*p* < 0.05	
	DGI	EATG	12.8 (6.4)	15.8 (6.6)	0.07 [−0.91, 1.05]^SMD^	*p* < 0.05	NSD
		ACG	13.3 (6.6)	15.3 (6.5)		*p* < 0.05	
Silkwood-Sherer (2007)	BBS	EATG	39.38 (16.87)	56 (15.11)	1.31 [0.04, 2.58]^†^	NR	*p*=0.048
		ICG	41.00 (9.19)	40.20 (7.91)		NR	
Lindroth (2015)	BBS	EATG	42 (1.73)	46 (0)	NA	NA	NA
Hammer (2005)	BBS	EATG	40.5 (24.57)	31 (26.06)	NA	NA	NA

*MOBILITY*							
Moraes (2021)	Timed up and go test	EATG	9.9 (3.1)	7.5 (2.2)	−0.27 [−0.95, 0.42]^SMD^	*p* < 0.001	*p*=0.398
		ICG	8.7 (2.5)	8.09 (2.13)		*p*=0.108	
Muñoz-Lasa (2011)	POMA	EATG	16 (6.1)	19.3 (3.6)	1.23 [0.38, 2.08]^†^	*p* < 0.005	NSD
		ACG	17.3 (6.8)	17.1 (6.7)		NSD	
Silkwood-Sherer (2007)	POMA	EATG	18.44 (6.45)	22.11 (4.82)	−2.05 [−3.51, −0.59]^†^	NR	*p*=0.078
		ICG	19.33 (3.9)	18.83 (3.98)		NR	
Hammer (2005)	Timed up and go test	EATG	14.85 (7.52)	14.82 (7.75)	NA	NA	NA

*SPASTICITY*							
Muñoz-Lasa (2019)	Modified Ashworth Scale	EATG	1.25 (0.25)	0.5 (0.55)	3.40 [1.11, 5.69]^†^	*p*=0.01	*p* < 0.0001
		ICG	1.12 (0.58)	0.82 (0.48)		NSD	
Vermöhlen (2017)	NRS	EATG	4.6 (2.1)	3.2 (2.4)	−0.25 [−0.74, 0.23]^SMD^	NR	*p*=0.031
		ICG	4.4 (2.2)	3.8 (2.3)		NR	

*WALKING PERFORMANCE*							
Moraes (2020)	Walking endurance (6-minute WT (m))	EATG	459.06 (118.34)	503.59 (126.38)	0.06 [−0.63, 0.74]^SMD^	*p*=0.144	*p* < 0.001
		ICG	513.00 (101.97)	497.13 (88.88)		*p* < 0.001	
Frevel (2015)	2-minute WT	EATG	130.3 (22.5)	141.3 (28.8)	0.24 [−0.75, 1.22]^SMD^	NSD	NSD
		ACG	128.6 (50.7)	130.0 (57.1)		NSD	

*GAIT SPEED*							
Moraes (2020)	Speed (cm/s)	EATG	97.84 (25.94)	114.93 (31.20)	0.29 [−0.39, 0.98]^SMD^	*p* < 0.001	*p* < 0.001
		ICG	110.95 (33.35)	105.95 (28.61)		*p*=0.095	
Muñoz-Lasa (2011)	Speed (m/s)	EATG	0.44 (0.11)	0.48 (0.10)	−0.38 [−1.2, 0.4]^d^		NSD
Lindroth (2015)	FGA	EATG	14 (4.36)	18 (6.24)	NA	NA	NA

*DISABILITY*							
Muñoz-Lasa (2011)	EDSS	EATG	5.2 (1.2)	5.2 (1.1)	0 [−0.8 , 0.8]^d^	NSD	NSD
		ACG	4.9 (1.3)	5 (1.3)	−0.08 [−0.8, 0.6]^d^	NSD	

*PAIN*							
Vermöhlen (2017)	VAS	EATG	32.2 (29.9)	24.9 (27.6)	0.05 [−0.43, 0.54]^SMD^	*p* < 0.05	NSD
		ICG	24.7 (29.3)	23.4 (27.0)		*p* < 0.05	

*PHYSICAL FUNCTIONING INDEPENDENCE LEVEL*							
Muñoz-Lasa (2011)	Barthel Index	EATG	89.6 (10.5)	90.4 (8.9)	−0.08 [−0.9, 0.7]^d^	NSD	*p*=0.055
		ACG	90.3 (10.9)	90.7 (11.3)	−0.02 [−0.8, 0.7]^d^	NSD	

*POSTURAL CONTROL*							
Moraes (2021)	CoP Speed (cm/s), stable surface, and eyes open	EATG	1.2 (0.4)	0.7 (0.4)	−1.21 [−1.96, −0.46]^SMD^	*p* < 0.001	*p*=0.004
		ICG	1.4 (0.7)	1.4 (0.7)		*p*=0.609	
	CoP Speed (cm/s), stable surface, and eyes closed	EATG	1.6 (0.6)	1.1 (0.6)	−0.98 [−1.70, −0.25]^SMD^	*p*=0.003	*p*=0.013
		ICG	2.0 (1.1)	1.7 (0.6)		*p*=0.078	
	CoP Speed (cm/s), foam surface, and eyes open	EATG	2.7 (0.9)	1.6 (0.9)	−0.80 [−1.51, −0.09]^SMD^	*p* < 0.001	*p*=0.019
		ICG	2.8 (1.1)	2.3 (0.8)		*p*=0.012	
	CoP Speed (cm/s), foam surface, and eyes closed	EATG	5.9 (2.2)	2.6 (1.6)	−1.14 [−1.88, −0.40]^SMD^	*p* < 0.001	*p* < 0.001
		ICG	6.4 (2.7)	5.10 (2.6)		*p*=0.005	

Menezes (2013)	AMPap (cm), eyes open	EATG	2.85 (0.93)	2.28 (0.68)	0.70 [−0.4, 1.8]^d^	*p* < 0.01	*p* < 0.01
		ICG	1.58 (0.35)	1.89 (0.99)	−0.42 [−1.8 , 1.0]^d^		
	AMPap (cm), eyes closed	EATG	3.91 (1.70)	3.02 (0.84)	0.66 [−0.4, 1.7]^d^	*p* < 0.01	*p* < 0.01
		ICG	2.39 (1.71)	2.61 (1.37)	−0.14 [−1, 5, 1.2]^d^		
	AMPml (cm), eyes open	EATG	2.2 (1.19)	1.66 (0.76)	0.54 [−0.5, 1.6]^d^	NSD	*p* < 0.01
		ICG	0.96 (0.43)	0.96 (0.63)	0 [−1.4, 1.4]^d^		
	AMPml (cm), eyes closed	EATG	3.28 (2.21)	2.17 (0.99)	0.65 [−0.4, 1.7]^d^	NSD	*p* < 0.01
		ICG	1.41 (0.67)	1.08 (0.67)	0.49 [−0.9, 1.9]^d^		
	Msap (cm/s), eyes open	EATG	1.44 (0.56)	1.48 (0.46)	−0.08 [−1.1, 1.0]^d^	NSD	*p* < 0.01
		ICG	0.83 (0.19)	0.97 (0.28)	−0.59 [−2.0, 0.8] ^d^		
	Msap (cm/s), eyes closed	EATG	1.91 (0.79)	1.85 (0.70)	0.08 [−1.0, 1.1]^d^	NSD	*p* < 0.01
		ICG	0.99 (0.27)	1.33 (0.35)	−1.09 [−2.6, 0.4]^d^		
	MSml (cm/s), eyes open	EATG	1.30 (0.75)	1.19 (0.59)	0.16 [−0.9, 1.2]^d^	*p*=0.02	*p* < 0.01
		ICG	0.62 (0.30)	0.72 (0.28)	−0.34 [−1.7, 1.1]^d^		
	MSml (cm/s), eyes closed	EATG	1.70 (0.99)	1.19 (0.41)	0.67 [−0.4, 1.8]^d^	*p*=0.02	*p* < 0.01
		ICG	0.69 (0.26)	0.84 (0.15)	−0.71 [−2.1, 0.7]^d^		

Lindroth (2015)	SOT	EATG	67.33 (9.29)	73 (12.49)	NA	NA	NA

SD: standard deviation; EATG: equine-assisted therapy group; ICG: inactive control group; NR: not reported; ACG: active control group; NSD: not significant differences; ^d^within group Cohen's d value with an interval confidence of 95%; ^SMD^Standardized Mean Difference results of randomized trials (calculated with after intervention data); ^†^Standardized Mean Difference results of nonrandomized trials (calculated with change from baseline data); NA: not applied; MSQOL-54: Multiple Sclerosis Quality of Life-54; HAQUAMS: Hamburg Quality of Life Questionnaire in Multiple Sclerosis; FAMS: Functional Assessment of Multiple Sclerosis Quality of Life; SF-36: Short Form 36; KHQ: general health perception of King's Health Questionnaire; FSS: Fatigue Severity Scale; MFIS: Modified Fatigue Impact Scale; VAS: Visual Analogue Scale; BBS: Berg Balance Scale; POMA: Performance Oriented Mobility Assessment; NRS: Numeric Rating Scale; WT: walking test; FGA: Functional Gait Assessment; EDSS: Extended Disability Status Scale; CoP: center of pressure; AMPap: amplitude of center of pressure displacement in the antero-posterior directions; AMPml: amplitude of center of pressure displacement in the medial-lateral directions; Msap: mean speed of the center of pressure displacement in the antero-posterior directions; MSml: mean speed of the center of pressure displacement in the medial-lateral directions; SOT: Sensory Organization Test.

**Table 4:** Risk of bias assessment.

Study	Item 1	Item 2	Item 3	Item 4	Item 5	Item 6	Item 7	Item 8	Total score
	Study design			Participants representativeness			Equivalence of comparison groups		
Moraes (2021)	Yes	Yes	Yes	Yes	No	Yes	Yes	Yes	7/8
Moraes (2020)	Yes	Yes	Yes	Yes	No	Yes	Yes	Yes	7/8
Muñoz-Lasa (2019)	Yes	Yes	Yes	No	No	Yes	Yes	No	5/8
Vermöhlen (2017)	Yes	Yes	Yes	Yes	No	Yes	Yes	Yes	7/8
Frevel (2015)	Yes	Yes	Yes	Yes	No	Yes	Yes	Yes	7/8
Lindroth (2015)	Yes	No	Yes	No	No	Yes	No	No	3/8
Menezes (2013)	Yes	Yes	Yes	No	No	Yes	Yes	Yes	6/8
Muñoz-Lasa (2011)	Yes	Yes	Yes	No	No	Yes	Yes	Yes	6/8
Silkwood-Sherer (2007)	Yes	Yes	Yes	No	No	Yes	Yes	Yes	6/8
Hammer (2005)	Yes	No	Yes	No	No	Yes	No	No	3/8

Item 1: cohort. Item 2: control or comparison group. Item 3: pre-/postintervention data. Item 4: random assignment of participants to the intervention. Item 5: random selection of participants for assessment. Item 6: follow-up rate of 80% or more. Item 7: comparison groups equivalent on sociodemographics. Item 8: comparison groups equivalent at baseline on outcome measures.

## Data Availability

The data supporting this systematic review are from previously reported studies and datasets, which have been cited.
